# Sperm telomere length as a novel biomarker of male infertility and embryonic development: A systematic review and meta-analysis

**DOI:** 10.3389/fendo.2022.1079966

**Published:** 2023-01-11

**Authors:** Yacheng Yuan, Yangyang Tan, Xiaolong Qiu, Hengfeng Luo, Yuxiang Li, Ruijie Li, Xukai Yang

**Affiliations:** ^1^ The First Clinical Medical College of Gansu University of Chinese Medicine, Lanzhou, China; ^2^ Department of Urology, The 940 Hospital of Joint Logistics Support Force of Chinese PLA, Lanzhou, China

**Keywords:** infertility, male, spermatozoa, telomere, embryos, diagnosis

## Abstract

**Background:**

Telomeres have an essential role in maintaining the integrity and stability of the human chromosomal genome and preserving essential DNA biological functions. Several articles have been published on the association of STL with male semen parameters and clinical pregnancy. The results, however, are either inconclusive or inconsistent. Therefore, this meta-analysis aimed to systematically assess the accuracy and clinical value of sperm telomere length (STL) as a new marker for diagnosing male infertility and predicting the quality of embryonic development.

**Methods:**

We performed a comprehensive systematic search for relevant publications in PubMed, the Cochrane Library, Web of Science, Embase, Scopus, and Ovid, from database build to August 2022. All experimental studies exploring the association of STL with male semen quality, male infertility, or embryonic development were included.

**Results:**

Overall, Twelve prospective observational cohort studies (1700 patients) were eligible for inclusion in the meta-analysis. The meta-analysis showed a positive linear correlation between STL and semen parameters. The optimal cut-off value for STL diagnosing male infertility was 1.0, with a sensitivity and specificity of 80%. Regarding STL and embryonic development, the clinical pregnancy rate was associated with longer STL, and there was no significant difference between the two groups regarding fertilization rate.

**Conclusion:**

Our study showed that STL has good diagnostic and predictive value for male fertility and clinical pregnancy and could be used as a new biomarker for diagnosing male infertility and predicting embryonic development.

**Systematic Review Registration:**

https://www.crd.york.ac.uk/PROSPERO/, identifier CRD42022303333.

## Introduction

The World Health Organisation defines sterility as “the inability to conceive successfully after more than 12 months of unprotected sexual intercourse”. It has been reported that over 50 million (approximately 15%) couples worldwide are affected (1). Male factors are involved in 51% of infertility problems (2, 3), of which up to 40% are diagnosed as idiopathic (4, 5). Currently, male fertility is mainly based on the initial assessment of semen analysis. The probability of conception depends on the quality of semen, which is reduced as one of the leading causes of male infertility (6), including reduced sperm concentration (oligospermia), decreased percentage of forward-moving sperm (weak sperm), a lower percentage of morphologically normal sperm (teratozoospermia), and complete absence of sperm in the semen (azoospermia) (7). However, basic diagnostic procedures using semen parameters are often inadequate to differentiate between fertile and infertile men (8). Among the approximately 30-40% of men with idiopathic infertility, standard semen parameters are often assessed in the ‘normal’ range (1, 9), and even when the semen analysis is below typical average values, it is not a direct indicator or predictor of fertility outcome (10). Hence, finding a new, non-invasive, reliable method to differentiate infertility from normal fertility is urgently needed.

Reactive oxygen species (ROS) are highly reactive oxidative radical, including superoxide anion radical ( ${\text{O}}_{2}^{•}$ ^−^), hydrogen peroxide (H_2_O_2_), nitric oxide (NO^•^), and hydroxyl radical (^•^OH) radicals, which in spermatozoa are mainly derived from activated leukocytes in the seminal plasma and the mitochondria (11). Due to the limited level of antioxidant defense of sperm, high levels of oxidative stress are highly susceptible to damage to sperm DNA and RNA transcripts, and extensive evidence suggests that reactive oxygen species-mediated sperm damage is a major cause of sperm damage in 30-80% of infertility patients (12). Telomeres are DNA-protein complexes located at the ends of chromosomes and consist of a non-coding hexamer formed by the tandem formation of a highly conserved repetitive DNA sequence (TTAGGG) forming a T-loop structure that interacts with the Shelterin protein complex to form a fully functional hooded structure (13). The specific structure allows cells to distinguish telomeres from sites of DNA damage, protects them from inappropriate DNA repair mechanisms, prevents gene degradation due to incomplete DNA replication, protects chromosome ends from erosion, and plays a crucial part in the integrity of the structure and stability of the chromosomal genome itself and preserving essential biological functions of DNA (14). Oxidative damage can disrupt telomere integrity and interfere with telomerase activity, leading to telomere shortening (15). The results of an *in vitro* test conducted by Lafuente et al (16)showed that the addition of hydrogen peroxide to sperm resulted in a reduction in measured sperm telomere length and a negative correlation between sperm telomere length and sperm reactive oxygen content (17). More recent studies have suggested that STL may be a promising marker of male reproductive biology (18). Most studies have concluded that STL is shorter in men with idiopathic infertility compared to fertile men. Telomere length is positively correlated with sperm anterograde motility and sperm count and negatively associated with sperm DNA fragmentation, and can be used as a marker of sperm quality (19–22). However, some scholars have suggested that telomere length shortening may be a sign of sperm damage rather than a cause of sperm alteration (23). In contrast, some studies have concluded that STL is unrelated to sperm parameters (24).

The telomere length has been attracting more and more attention in the reproductive field. Several studies have shown a greater preponderance of telomere length and telomerase activity in the cumulus cells (25), granulosa cells (26), and peripheral lymphocytes (27) of fertile women compared to infertile women. Nevertheless, limitations in the collection of material, particularly cumulus cells, make it challenging to apply these findings to clinical predictions of embryonic developmental quality and pregnancy outcome. Compared to cumulus cells and other cells, semen samples are easier to collect and assay. The telomere length of sperm cells typically correlates positively with the high-quality and transferable embryo ratio (28). In addition, the incidence of sustained pregnancy after *in vitro* fertilization (IVF) treatment among patients with relatively abnormal STL was zero, compared to 35.7% in samples with STL in the normal range, which may indicate that STL plays an essential role in reproduction (29). Whether STL is recommended as a diagnostic and predictive clinical outcome for male infertility remains controversial. To date, there have been no meta-analyses to assess the value of STL in the field of reproduction. In this study, we comprehensively analyzed and evaluated the current studies on STL concerning male infertility and embryonic development to clarify STL’s accuracy and clinical value in diagnosing male infertility and predicting embryonic developmental quality.

## Materials and methods

The study is transparent and original, adhering to the Cochrane Manual version 6.2 and Preferred Reporting Items for Systematic Reviews and Meta-Analyses (PRISMA) guidelines on systematic reviews and meta-analyses (30, 31). Systematic reviews and meta-analyses should be registered to avoid publication bias (32). Therefore, we have completed registration with the International Prospective Register of Systematic Reviews (PROSPERO) under the registration ID CRD42022303333. As this study involved only the collection and collation of clinical study data, institutional review board (IRB) approval was not required.

### Search strategy

We performed a comprehensive systematic search for relevant publications in PubMed, the Cochrane Library, Web of Science, Embase, Scopus, and Ovid, from database build to August 2022. The search formula was the following terms: (((sperm telomere length) OR (telomere length)) OR (STL)) AND (((((Male Infertility) OR (Sub-Fertility Male)) OR (Embryo)) OR (embryonic development)) OR (Pregnancy)) and further consulted their references to expand the search without language and year restrictions.

### Study selection and data extraction

Inclusion criteria (1): all experimental studies exploring the association of STL with male semen quality or male infertility or embryonic development (2); the diagnosis of infertility or subfertility included all degrees of altered semen parameters (except azoospermia) (3); the study reported at least one extractable outcome such as sperm count, percentage of forward-moving sperm (a+b%) and sperm concentration.

**Figure 1:**
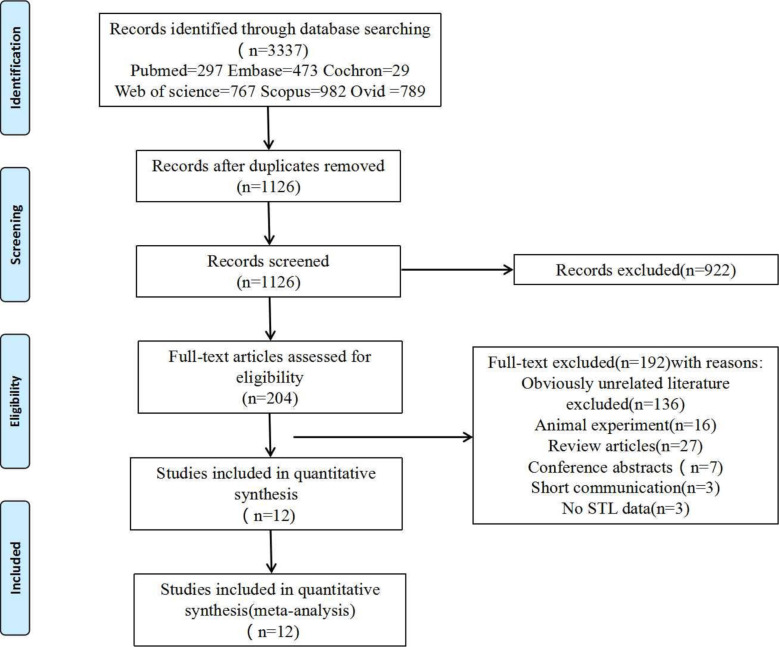
Flow of studies through the review.

Exclusion criteria (1): Reviews, conference abstracts, or duplicate publications (2). Insufficient data or inability to download the full text (3). The subject of the literature is not human. The specific reasons excluded are illustrated in **Figure 1**. two independent reviewers (X.Q and H.L) screened the searched literature to remove duplicates and find the full text of the articles. When disagreements arise, a consensus is reached through thorough discussion and analysis with a third reviewer (X.Y).

Extract data of interest from each included study. Include authors, publication year, study region, type of study design, experimental method, inclusion or exclusion criteria, and the number of subjects. The assessment of STL was used as the primary endpoint for inclusion in the literature search. In addition, (X.Q., H.L., and X.Y.) separate quality checks were performed on the extracted data.

### Assessment of methodological quality

The investigators (Qiu and Luo) applied the Cochrane risk of bias tools to assess the risk of bias for all included studies independently. Review manager 5.3 was used to represent the risk of bias assessment results visually. The assessment was based on the following quality assessment criteria: I Randomisation methods, although not an inclusion criterion; II Concealed allocation; III Double-blinding process for subjects and staff; IV Participants recruited, number of analyzed or dropped from the track; V Selective reporting VI Other risks of bias.

### Statistical analysis

Data analysis using Review Manager 5.3 software. All of the papers included in this study are observational and prospective cohort studies, with weighted mean differences (WMD) applied to continuous variables and relative risk ratios (RR) applied to dichotomous variables, and all with their 95% confidence intervals (CI) as effect indicators. The I^2^ test to analyze heterogeneity across studies, Choice of effect model based on the size of heterogeneity (fixed-effects model when P>0.1 or I^2^<50%; random-effects model when P ≤ 0.1 or I^2^>50%). An IBM SPSS Statistics 25.0 (SPSS Inc., Chicago, IL, U.S.A.) software was used to plot to scatter plots to test the correlation between STL and semen parameters, thus further validating the statistical results. The Receiver operating characteristic curve (ROC) was applied to test the diagnostic accuracy of STL for male infertility. The Uden index was used to calculate the ROC threshold to determine the best pairing of sensitivity and specificity to identify better the best cut-off value for diagnosing infertile men. Meanwhile, the Hosmer and Lemeshow tests were applied for goodness-of-fit to test the working of the scoring model. A sensitivity analysis was finally performed to identify sources of heterogeneity, using Review Manager 5.3 funnel plots tools to assess publication bias. If P<0.05 is considered to be statistically significant.

## Results

### Literature search

In total, literature searches identified 3,337 papers, and 12 articles (10, 18, 29, 33–41) were finally included after excluding duplicate studies, irrelevant literature, review articles, and short communications adhering strictly to the inclusion and exclusion criteria. Details of the screening are illustrated in **Figure 1**.

### Characteristics and qualitative results of included studies

Overall, a total of 887 fertile men (control group) and 813 infertile men (experimental group) were compared in the 12 studies finally included in the meta-analysis, including patients from Italy, Spain, Portugal, the UK, Iran, China, and India. The publication dates of the literature varied from 2012 to 2021, and the sample sizes changed from 10 to 345 patients. The primary characteristics and study features of the included studies are listed in **Table 1**. The quality of each study was calculated using the Cochrane Risk of Bias Assessment Tool, with studies categorized as ‘low risk, ‘high risk,’ or ‘unclear risk. Six of the 12 studies had a high risk for at least one department, and Low bias risks accounted for 83.3% of all departments. Ultimately, a low overall risk of bias was revealed in (**Supplementary Figure 1**).

**Table 1:** Characteristics of studies included in the analysis.

					STL	Fertile men(CONTROL GROUP)			Infertile men(STUDY GROUP)		
Author	N.R	Country	Year	Study design	Method	N	Age(years)	Inclusion criteria	N	Age(years)	Inclusion criteria
Lopes, AC. et al. (10),	68	Portugal	2020	O.C.S. (P)	qRT-PCR	33	39.3 ± 4.1	normozoospermic	45	39.3 ± 4.1	non-normozoospermic
Cariati, F. et al. (29),	45	England	2016	O.C.S. (P)	qRT-PCR	54	39.4 ± 5.5	normozoospermic	19	39.3 ± 5.3	oligozoospermic
Ferlin, A. et al. (33),	35	Italy	2013	O.C.S. (P)	qRT-PCR	61	Range 18-19	normozoospermic	20	Range 18-19	oligozoospermic
Thilagavathi, J. et al. (18),	19	India	2012	O.C.S. (P)	qRT-PCR	25	N.A.	Proven fertility	32	N.A.	Unexplained infertility
Liu, SY. et al. (34),	18	China	2015	O.C.S. (P)	qRT-PCR	138	Range 22-52	Proven fertility	126	Range 23-57	Unexplained infertility
Torra-Massana, M. et al. (35),	27	Spain	2018	O.C.S. (P)	qRT-PCR	60	24.3 ± 5	Positive	60	24.3 ± 5	Negative
Rocca, MS. et al. (36),	44	Italy	2021	O.C.S. (P)	qRT-PCR	30	36.1 ± 6.8	Proven fertility	35	39.0 ± 6 5.4	Oligozoospermic normozoospermia
Amirzadegan, M. et al. (37),	30	Iran	2021	O.C.S.(P)	qRT-PCR	10	40.3 ± 3.75	Proven fertility	10	35.46 ± 5.59	oligozoospermic
Mishra, S. et al. (38),	31	India	2016	O.C.S.(P)	qRT-PCR	102	32.2 ± 4.0	Proven fertility	112	31.71 ± 4.45	Unexplained infertility
Yang, Q. et al. (39),	54	China	2016	O.C.S. (P)	qRT-PCR	345	30.4 ± 4.0	Positive	306	30.5 ± 3.9	Negative
Darmishonnejad, Z. et al. (40),	59	Iran	2019	O.C.S.(P)	qRT-PCR	10	40.11 ± 3.14	Proven fertility	10	38.10 ± 4.17	Unexplained infertility
Darmishonnejad, Z. et al. (41),	44	Iran	2020	O.C.S. (P)	qRT-PCR	19	40.47 ± 3.82	Proven fertility	38	32.65 ± 6.56	Unexplained infertility

N.R, number of references; O.C.S., observational clinical study; P, prospectively collected data; qRT-PCR, Quantitative Real-Time Polymerase Chain Reaction; N.A., not available; Positive the longer STL, Negative the shorter STL.

### Semen parameters

Sperm count data were available from seven studies (10, 29, 34, 36–38, 40). The mean sperm count was (160.64 ± 149.32) × 10^6^/per ejaculation in the fertile group and (94.82 ± 103.55) × 10^6^/per ejaculation in the infertile group. Meta-analysis indicated that the sperm count of the fertile group was superior as compared to the infertile group (WMD 2.73, 95% CI:1.70-3.76, I^2^ = 96%, p < 0.00001), The difference was statistically significant (**Supplemental Figure 2**).

Seven studies provided data on the percentage of forwarding motile sperm (a+b) % (10, 29, 34, 36, 37, 40, 41). The Meta-analysis results suggested that the rate of forward-moving sperm (a+b) % was significantly better in the fertile individuals (WMD 3.17, 95% CI: 1.84-4.51, I^2^ = 96%, p < 0.00001), The difference was statistically significant (**Supplemental Figure 3**).

Six studies provided data on sperm concentration (10, 34, 36, 37, 40, 41). The mean sperm concentration was (88.67 ± 54.89) ×10^6^/ml in the fertile group and (48.50 ± 39.67) ×10^6^/ml in the infertile group. As Meta-analysis revealed, Sperm concentrations were likewise visibly higher in the fertile group (WMD 2.77, 95% CI:1.52-4.02, I^2^ = 96%, p < 0.00001), The difference was statistically significant (**Supplemental Figure 4**).

Four out of twelve studies of this analysis report sperm DNA fragmentation value obtained using TUNEL analysis (29, 37, 40, 41). The mean Sperm DNA Fragmentation Index was significantly higher in infertile men (25.96 ± 10.42)% than in fertile individuals (20.98 ± 10.45)%. As Meta-analysis revealed, sperm DNA fragmentation value was likewise visibly more increased in the infertile group (WMD 6.89, 95% CI:3.52-10.26, I^2^ = 94%, p < 0.0001), The difference was statistically significant (**Supplemental Figure 5**).

### Sperm telomere length (STL)

**Figure 2:**
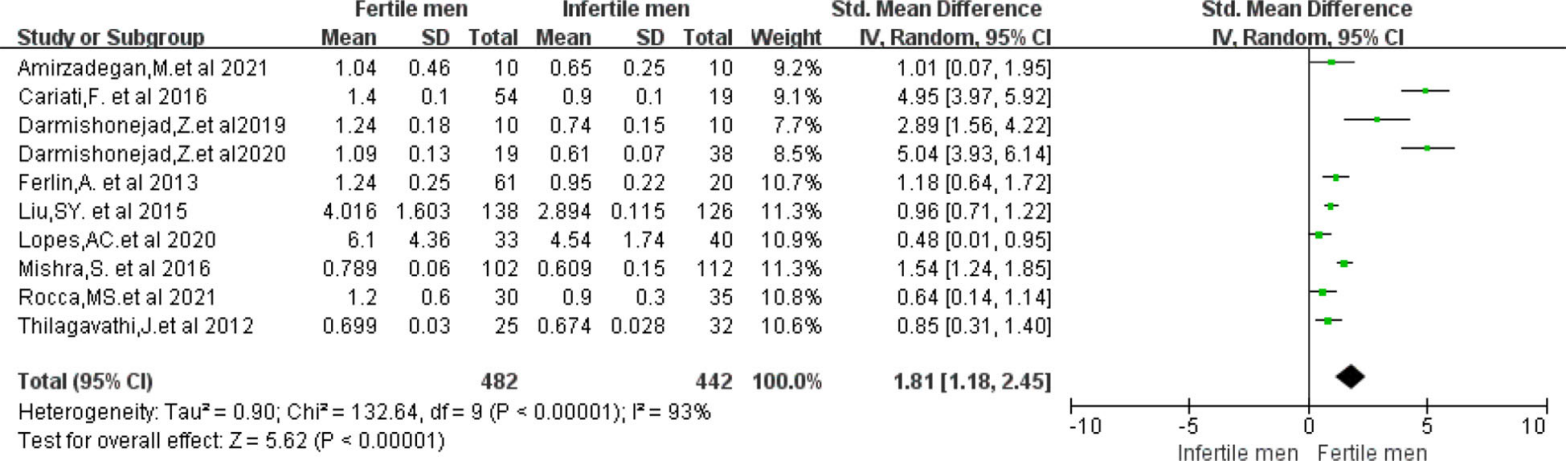
Forest plot showing the results of Meta-analysis, comparing fertile and infertile men for differences in STL.

STL data were available from the 10 included studies (10, 18, 29, 33, 34, 36–38, 40, 41). The mean STL was (2.24 ± 2.21) in the fertile group of men and (1.67 ± 1.43) in the infertile group. In the Meta-analysis, the fertile group had a higher STL than the infertile group (WMD 1.81, 95% CI:1.18-2.45, I^2^ = 93%, p < 0.00001), and the difference was statistically significant (**Figure 2**). Furthermore, when comparing the five studies that included men of proven fertility with Unexplained infertility (18, 34, 38, 40, 41), the results remained significantly different (WMD 2.05, 95% CI:1.21-2.90, I^2^ = 93%, P < 0.00001) (**Supplemental Figure 6**).

### Correlation between STL and semen parameters

There might be a correlation between STL and semen analysis parameters, as indicated by the results of the Meta-analysis. Therefore, separate scatter plots were drawn to demonstrate a significant positive linear correlation between STL and semen parameters: a higher sperm count (R^2^ = 0.162, P=0.154) (**Supplemental Figure 7**), percentage of forward-moving sperm (a+b)% (R^2^ = 0.033, P=0.501) (**Supplemental Figure 8**), sperm concentration (R^2^ = 0.037, P=0.549) (**Supplemental Figure 9**) resulted in a longer STL.

### Male infertility diagnosis

**Figure 3:**
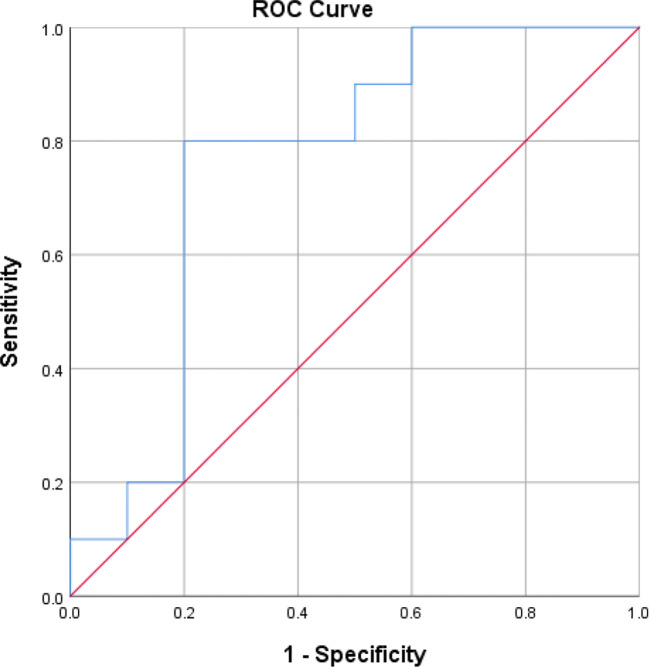
The Receiver operating characteristic(R.O.C.) curve for determining the optimal STL cut-off value for the diagnosis of male infertility.

ROC curves ([AUC]=0.76, p<0.05) for all data from the 12 included studies for STL. The optimum cut-off value for the STL was ascertained by calculating the Jorden index for the best pairing of sensitivity and specificity, which was 1.0. At this threshold, the diagnostic ability of STL showed a sensitivity and specificity of 80% (**Figure 3**). The Hosmer and Lemeshow tests were also applied for the goodness of fit (P=0.40, P>0.05) (**Supplemental Figure 10**). Our outcomes demonstrate that the combined sensitivities in this study were generally good and that the scoring model worked well.

### Embryonic development

A total of 3 studies provided data on fertilization rates (10, 39, 40). Meta-analysis was performed in 3 studies with Considerable heterogeneity (I^2^ = 83%), and a random effects model was applied. Our results revealed that higher STL groups did not show a clear advantage in fertilization rates with no statistically meaningful difference(RR=0.99; 95% CI:0.86-1.14; P=0.88) (**Supplemental Figure 11**).

**Figure 4:**
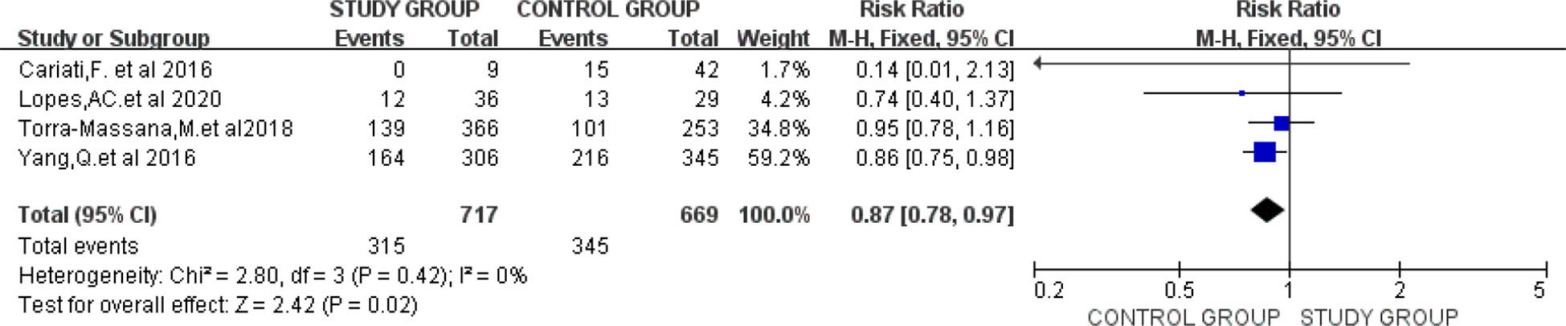
Forest plot showing the results of Meta-analysis, comparing study and control men for differences in clinical pregnancy rates.

Four studies provided data on clinical pregnancy rates (10, 29, 35, 39). Meta-analysis was performed in the four studies with low heterogeneity between studies (I^2^ = 0%), and a fixed effects model was applied. Our results revealed that higher STL groups possessed superior clinical pregnancy rates and the two groups showed statistically significant differences (RR=0.87; 95% CI:0.78-0.97; P=0.02) (**Figure 4**).

### Sensitivity analysis and publication bias

A sensitivity analysis was undertaken using the exclusion of individual studies from the outcome analysis to assess whether individual studies would affect the overall results. Meta-analysis results were similar after excluding each study, validating the stability of the Meta-analysis. When the three studies by Cariati F and Darmishonnejad Z (29, 40, 41) were excluded, the recalculated results showed a marked reduction in heterogeneity (WMD 0.97, 95% CI:0.67-1.28, I^2^ = 69%, p<0.00001). Using STL as an indicator by funnel plots, we found no significant publication bias (**Supplemental Figure 12**).

## Discussion

Research on STL has recently increased yearly, with more scholars focusing on its role in human reproduction. Although several studies have been previously published on the relationship between STL and male semen and embryos, this is the first study to systematically review and meta-analyze the value of STL as a new biomarker for diagnosing male infertility and predicting embryonic development, filling a gap in the field. Our study confirms a clear positive correlation between STL and semen quality parameters and influences embryonic development and clinical pregnancy outcome, which could be considered a new diagnostic tool for diagnosing male infertility and predicting the quality of embryonic development, adding more definitive information to conventional semen analysis.

Male infertility is a global disease that threatens human development with genetic characteristics, and the incidence of male infertility is rising worldwide (42, 43). A meta-analysis showed a 50% to 60% reduction in male sperm count compared to 40 years ago (44). In most cases, clinical patients are found to be infertile only after marriage, but by then, the best treatment and male fertility time may have been missed. In severe cases, this can lead to a decline in marital quality and affect family well-being (45). However, to date, there are still no laboratory indicators for specific early diagnosis, standard semen analysis does not accurately distinguish between fertile and infertile populations, and diagnostic confusion often exists, as confirmed by the studies we included (18, 34, 36, 38, 40, 41). Therefore, searching for the ideal biomarker is essential in the early diagnosis, treatment, and prognosis of male infertility.

Telomeres protect chromosome ends from erosion and maintain human gametogenesis and fertility (46). The telomere function is largely limited by its length. Once telomere shortening exceeds a critical level, the proteins forming the Shelterin complex are unable to bind to telomeric sequences and cannot perform capping at chromosome ends (47). Therefore, it is essential to maintain telomere length, which is generally achieved by telomerase, a ribonucleoprotein complex consisting of a unique telomerase reverse transcriptase (TERT) and telomerase RNA (TERC) that synthesizes new telomeric repeats by copying its telomerase RNA component copy extension 3’ end (48). In humans, telomerase is present in germ cells, stem cells, and about 85% of cancer cells. It is particularly active in germ cells for extended periods, thereby delaying telomere erosion and avoiding chromosomal segregation defects such as aneuploidy or gamete imbalance (49). Due to the delayed closure of telomerase, sperm telomeres are generally longer than somatic cells, with a length of about 10-20 Kb (14). Inactivation of telomerase leads to progressive telomeres shortening, which shorten by about 40-200 bp base pairs with each cell division until they reach a specific limit when the cell stops dividing and dies of senescence (50). Three essays are commonly used in studies to determine STL. The southern blot is mainly used as a reference method to validate newly imported technologies (51); q-fish can only be used for mitotically active cells (52). In contrast, only Q-PCR can be performed in isolated DNA (53). The Q-PCR method was used to measure STL in the literature included in this study. We speculate that this may have increased the risk of bias and limited the predictive strength of the STL cut-off values obtained from the survey to some extent. However, the results were conclusive in the studies that used the Q-PCR method. The Q-PCR method is more suitable for epidemiological studies in large populations due to its relative simplicity, affordability, and ability to use smaller amounts of DNA. It is also used in clinical practice to measure STL in most cases. Therefore, we are still recommending this cut-off value and look forward to additional studies in the future to determine a more comprehensive outcome.

Telomere attrition is an inevitable and normal biological event during cellular aging. Apart from progressive telomere shortening due to cell division problems, telomere length is still influenced by many factors such as age, genetics, environment, and psychosocial stress levels (49, 54, 55). In Ferlin’s study (33), by including 61 patients with normospermia and 20 patients with idiopathic oligospermia, it was found that older fathers and mothers had longer STL in their offspring and that STL was directly related to the age of the parents at the time of pregnancy. Still, the relative contribution of paternal and maternal age could not be determined. In certain genetic disorders, abnormal telomere shortening is caused by gene dysregulation that disrupts telomeres’ integrity and stability, known as telomeropathies (56). Although no specific mechanisms linking telomeres to the pathogenesis of these diseases have been identified, it has been shown that most of them exacerbate pathological conditions associated with aging, such as cardiovascular disease and diabetes (57). The adverse effects of environmental pollution on telomere length have been confirmed by a systematic review of 12,058 subjects (55), which showed a direct link between air pollution exposure and shortened telomere length. Increased levels of oxidants and poor lifestyles also contribute to telomere erosion (58). On the other hand, humans can reduce telomere erosion through physical activity (59). One meta-analysis of the association between diet and telomere length maintenance showed that the Mediterranean diet protects telomere integrity through its anti-inflammatory and antioxidant properties (60). It has been found that the physiological process of telomere attrition may be accelerated under certain pathological conditions. A recent study of 38 infertile men and 19 fertile men reported that a comparative analysis of STL and semen quality in male patients found that infertile men generally had shorter STL than fertile men (41). Several meta-analyses have previously reported that sperm DNA fragmentation indices can influence fertilization, embryonic development, and pregnancy outcome (61, 62), and a high proportion of DNA fragmentation was found in sperm from men with shorter telomeres (63). This finding further supports the correlation between STL and male fertility. Our results showed that infertile men had a significantly higher sperm DNA fragmentation index than fertile men, which is consistent with the findings of previous studies. Animal studies have also found that long telomeres are only inherited in male mice whose parents have longer telomeres (64) and that longer telomeric sperm can lead to higher rates of morulae and blastocysts (65). In some studies (20, 22, 33, 40), a comparative analysis of the relationship between STL and semen quality in infertile male patients suggested that telomeres could be considered as a biomarker of abnormal spermatogenesis quality and quantity. Our analysis also showed that fertile men had a higher STL in comparison to infertile men (2.24 ± 2.21 vs. 1.67 ± 1.43, p < 0.001), and STL was significantly associated with sperm count (R^2^ = 0.162), percentage of forward-moving sperm (a+b) % (R^2^ = 0.033) and sperm concentration (R^2^ = 0.037). A positive linear correlation was calculated, and statistical analysis showed that the Cut-off value = 1.0 could be used to predict male fertility, the same as the results of several previous studies.

A controversial viewpoint: STL may be able to predict successful implantation and embryo quality after assisted reproductive treatments (ART), such as *in vitro* fertilization (IVF), among infertile couples (21). Selecting sperm with longer telomeres facilitates the production of better-quality embryos and may influence pregnancy outcomes and the success of ART (66). However, some scholars have argued that STL is not helpful in predicting the outcome of intracytoplasmic sperm injection therapy (ICSI) (35), with no significant correlation between STL and clinical outcome. Two different conclusions may be explained by the fact that part of the study did not relate male STL to the physical characteristics of the female partner. Telomeres have been reported to be shorter in the oocytes of women who are not pregnant after IVF (67), and telomere length may limit the ability of fertilized eggs to develop into healthy embryos. Indeed, many complications of advanced age are associated with shorter embryonic telomere lengths, including Down’s syndrome (68) and recurrent miscarriage (69). A recent meta-analysis (70) that included 105 studies involving 271,632 pregnant women suggested that higher body mass index (BMI) values may indicate poor pregnancy outcomes and that higher BMI is linearly associated with higher miscarriage rates, lower clinical pregnancy rates and lower live birth rates. Therefore, certain variables (e.g., female age, BMI, embryo morphology, etc.) should be included in future ART studies. The results of this study showed clinical pregnancy rates associated with longer STL (RR=0.87; 95% CI:0.78-0.97), and two groups did not show a large difference in fertilization rates (RR=0.99; 95% CI:0.86-1.14). This may be due to a smaller sample size and more significant heterogeneity between studies. Notably, our findings clarify the relevance of STL to clinical pregnancy outcomes and herald a potentially crucial mechanistic role in embryonic development. A recent study suggests that the increased risk of IVF failure and recurrent miscarriage may be associated with embryonic aneuploidy. Short telomeres significantly cause increased aneuploidy abnormalities and delayed embryo development (25). Thus, we hypothesize that STL may be a promising predictor of embryonic developmental quality, both for natural conception and IVF, as it may reflect embryonic quality to some extent and predict pregnancy success.

Our meta-analysis has several advantages over existing published meta-analyses. First, our meta-analysis is the first systematic assessment of STL in diagnosing male infertility and predicting embryonic developmental quality. Secondly, we applied the area under the ROC curve and the Hosmer and Lemeshow test to a comprehensive test of the accuracy and feasibility of STL in diagnosing male infertility, providing a comprehensive assessment of its clinical diagnostic ability. However, the study still showed several limitations: firstly, the inclusion criteria were not homogeneous, and there were significant individual differences between the patients enrolled in the study, resulting in heterogeneity between studies, which needs to be further eliminated in future studies by rationalizing the design and increasing the sample size. In addition, subgroup analysis of the assay was not performed, leading to a higher likelihood of false positive or negative results.

## Conclusions

In conclusion, STL has diagnostic and predictive value for males in fertility and clinical pregnancy. In conjunction with the specific clinical situation, it may be possible in the future to combine tests with other biomarkers in the clinic, such as the combined testing of semen parameters and sperm DNA fragmentation index, thus further improving the diagnostic sensitivity and specificity of male infertility and the ability to assess and predict pregnancy outcomes, which will play a vital role in the future diagnosis and treatment of human reproductive disorders.

## Data availability statement

The original contributions presented in the study are included in the article/**Supplementary Material**. Further inquiries can be directed to the corresponding author.

## Author contributions

YY was a significant contributor to the writing of the manuscript, contributed to the design and data acquisition of the study, drafted and critically revised the article, and organized the final approval of the version to be published. YT contributed to the study concept and design, data acquisition, and data analysis and interpretation. XQ, HL, YL, and RL assessed the quality of the included studies and critically revised the manuscript for important intellectual content. XY had full access to all of the data in the study and agreement to be accountable for all aspects of the work in ensuring that questions related to the accuracy or integrity of any part of the work are appropriately investigated and resolved. All authors contributed to the article and approved the submitted version.
