# Supplementary material for: Sperm telomere length as a novel biomarker of male infertility and embryonic development: A systematic review and meta-analysis
Source: Front Endocrinol (Lausanne). 2023 Jan 11;13:1079966. doi: 10.3389/fendo.2022.1079966 (PMC9875015; doi:10.3389/fendo.2022.1079966)
Supplement: Supplementary file 1 [file DataSheet_1.docx]

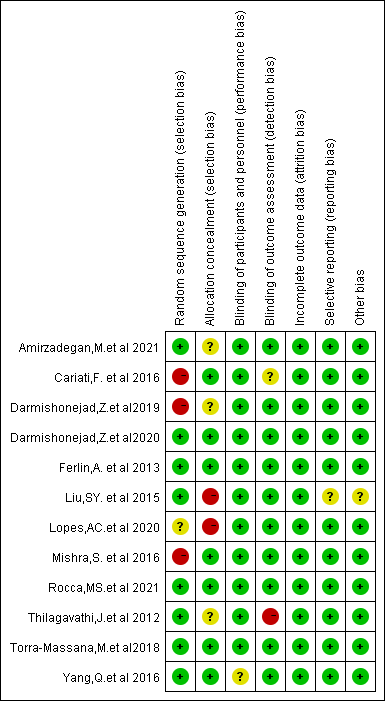


Supplementary Figure 1. Risk of bias for included studies


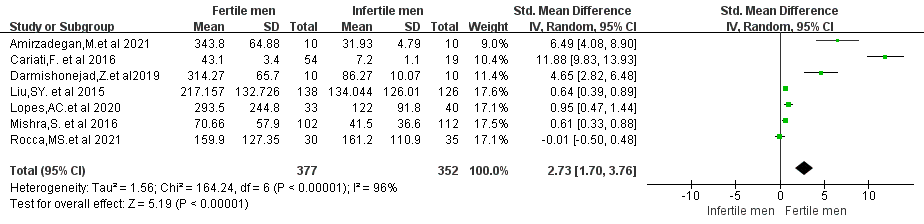


Supplementary Figure 2. Forest plot showing the results of Meta-analysis, comparing fertile and infertile men for differences in sperm count.


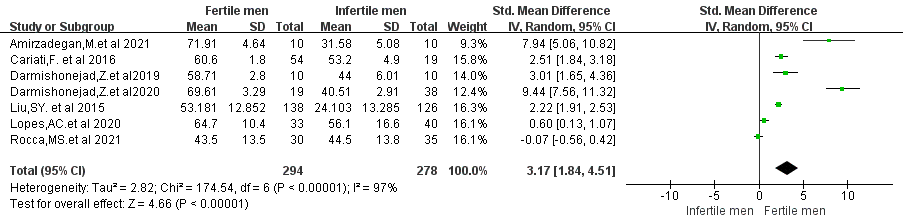


Supplementary Figure 3. Forest plot showing the results of Meta-analysis, comparing fertile and infertile men for differences in Percentage of forward-moving sperm (a+b) %.


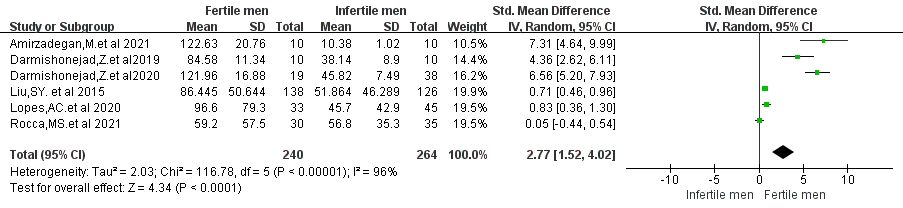


Supplementary Figure 4. Forest plot showing the results of Meta-analysis, comparing fertile and infertile men for differences in sperm concentration.


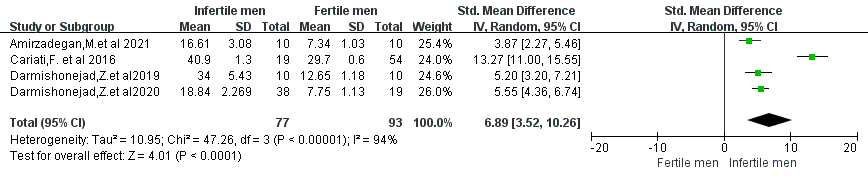


Supplementary Figure 5. Forest plot showing the results of Meta-analysis, comparing fertile and infertile men for differences in sperm DNA fragmentation.


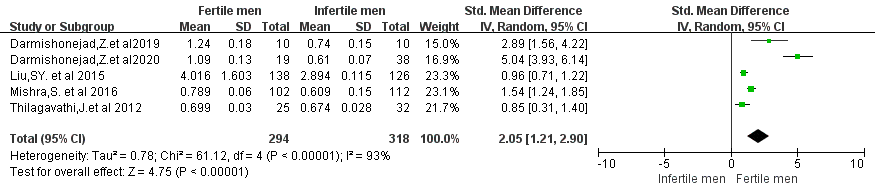


Supplementary Figure 6. Forest plot showing the results of Meta-analysis, comparing proven fertility and Unexplained infertility men for differences in STL.

**
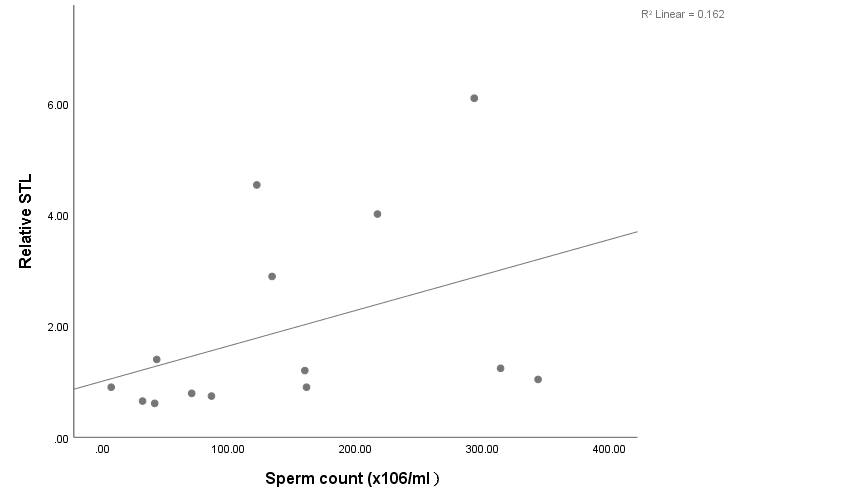
**

Supplementary Figure 7. Separate scatter plots of the correlation results between STL and sperm count.


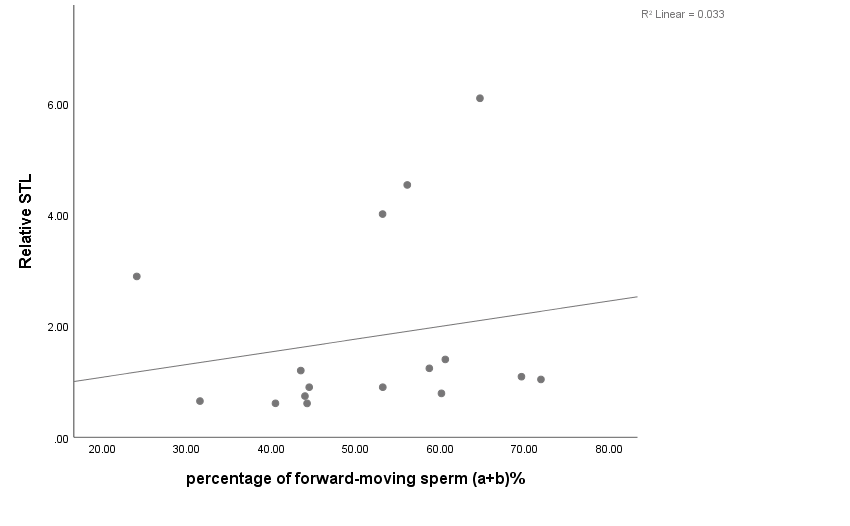


Supplementary Figure 8. Separate scatter plots of the correlation results between STL and Percentage of forward-moving sperm (a+b)%.

**
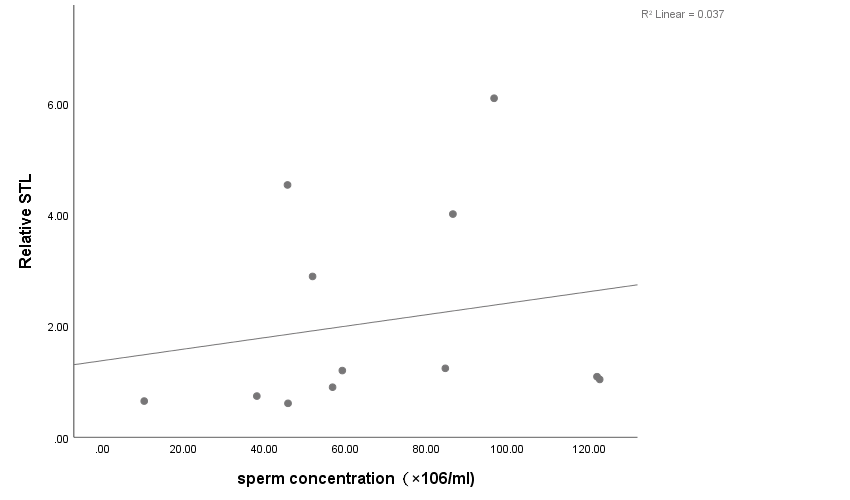
**

Supplementary Figure 9. Separate scatter plots of the correlation results between STL and sperm concentration.


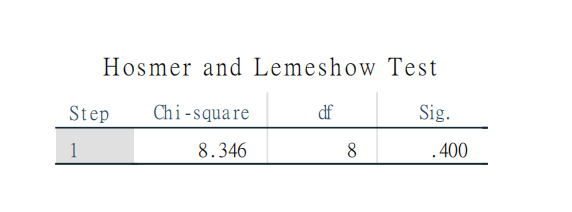


Supplementary Figure 10. Goodness-of-fit tests for evaluating the working effectiveness of the scoring model


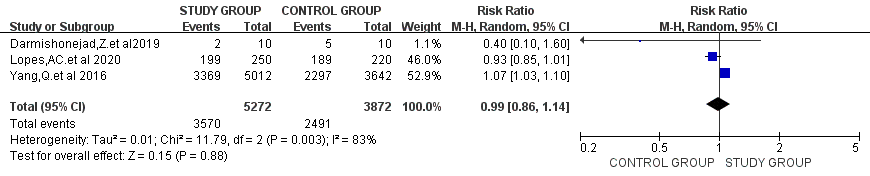


Supplementary Figure 11. Forest plot showing the results of Meta-analysis, comparing study and control men for differences in fertilization rates.


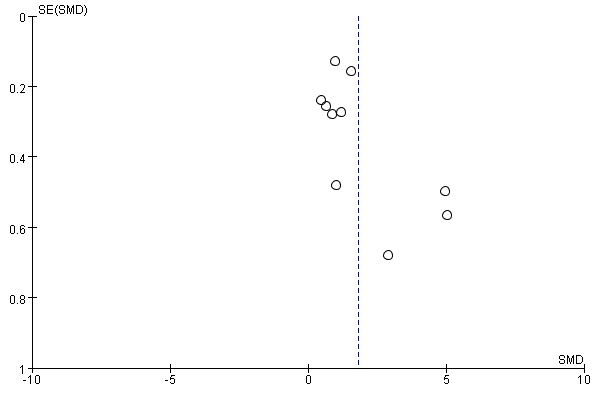


Supplementary Figure 12. Funnel plot demonstrating results of publication bias for STL-specific assessments
